# High-Level Patchoulol Biosynthesis in *Artemisia annua* L.

**DOI:** 10.3389/fbioe.2020.621127

**Published:** 2021-02-04

**Authors:** Xueqing Fu, Fangyuan Zhang, Yanan Ma, Danial Hassani, Bowen Peng, Qifang Pan, Yuhua Zhang, Zhongxiang Deng, Wenbo Liu, Jixiu Zhang, Lei Han, Dongfang Chen, Jingya Zhao, Ling Li, Xiaofen Sun, Kexuan Tang

**Affiliations:** ^1^Joint International Research Laboratory of Metabolic & Developmental Sciences, Key Laboratory of Urban Agriculture (South) Ministry of Agriculture, Plant Biotechnology Research Center, Fudan-Shanghai Jiaotong University (SJTU)-Nottingham Plant Biotechnology R&D Center, School of Agriculture and Biology, Shanghai Jiao Tong University, Shanghai, China; ^2^Southwest University-Tibet Agriculture and Animal Husbandry College (SWU-TAAHC) Medicinal Plant Joint R&D Centre, School of Life Sciences, Southwest University, Chongqing, China; ^3^Corporate R&D Division, Firmenich Aromatics (China) Co. Ltd., Shanghai, China

**Keywords:** patchoulol, *Artemisia annua* L., synthetic biology, sesquiterpenoids, terpenes

## Abstract

Terpenes constitute the largest class of secondary metabolites in plants. Some terpenes are essential for plant growth and development, membrane components, and photosynthesis. Terpenes are also economically useful for industry, agriculture, and pharmaceuticals. However, there is very low content of most terpenes in microbes and plants. Chemical or microbial synthesis of terpenes are often costly. Plants have the elaborate and economic biosynthetic way of producing high-value terpenes through photosynthesis. Here we engineered the heterogenous sesquiterpenoid patchoulol production in *A. annua*. When using a strong promoter such as 35S to over express the avian farnesyl diphosphate synthase gene and patchoulol synthase gene, the highest content of patchoulol was 52.58 μg/g DW in transgenic plants. When altering the subcellular location of the introduced sesquiterpene synthetase via a signal peptide, the accumulation of patchoulol was observably increased to 273 μg/g DW. This case demonstrates that *A. annua* plant with glandular trichomes is a useful platform for synthetic biology studies.

## Introduction

Plants synthesize and secrete a good deal of secondary metabolites, some of which are considerable, economically in industry, agriculture, and pharmaceuticals (Balandrin et al., 1985; Pichersky and Gershenzon, 2002). Terpenes comprise the largest class of secondary metabolites in plants (Kappers et al., 2008). Many of them, such as phytohormones (abscisic acid, brassinosteroid, and gibberellin), sterols and carotenoid pigments, play critical roles in plant growth, development, membrane components, and photosynthesis (Bohlmann and Keeling, 2008). In addition, the majority of plant terpenes are involved in the interaction of plant with the environment and other organisms (Gershenzon and Dudareva, 2007). For instance, some terpenes bear antibacterial and antifungal activity (Rastogi et al., 1998; Lunde and Kubo, 2000). They can also hold the protective role in plants defense system against insects, mollusks, fish and nematodes (Lorimer et al., 1996; Ito et al., 1997; Laurent et al., 2003; Quintana et al., 2003). Terpenes can sometimes act a tool of communication among organisms. For instance, when the predators attack aphids, they normally release a kind of terpenoid, (*E*)-β-farnesene, as an alarm pheromone, to disperse and leave the host. Besides, (*E*)-β-farnesene is also released to attract natural enemies of aphids at the same time in plants (Hardie and Minks, 1999; Kunert et al., 2005).

**Figure 1:**
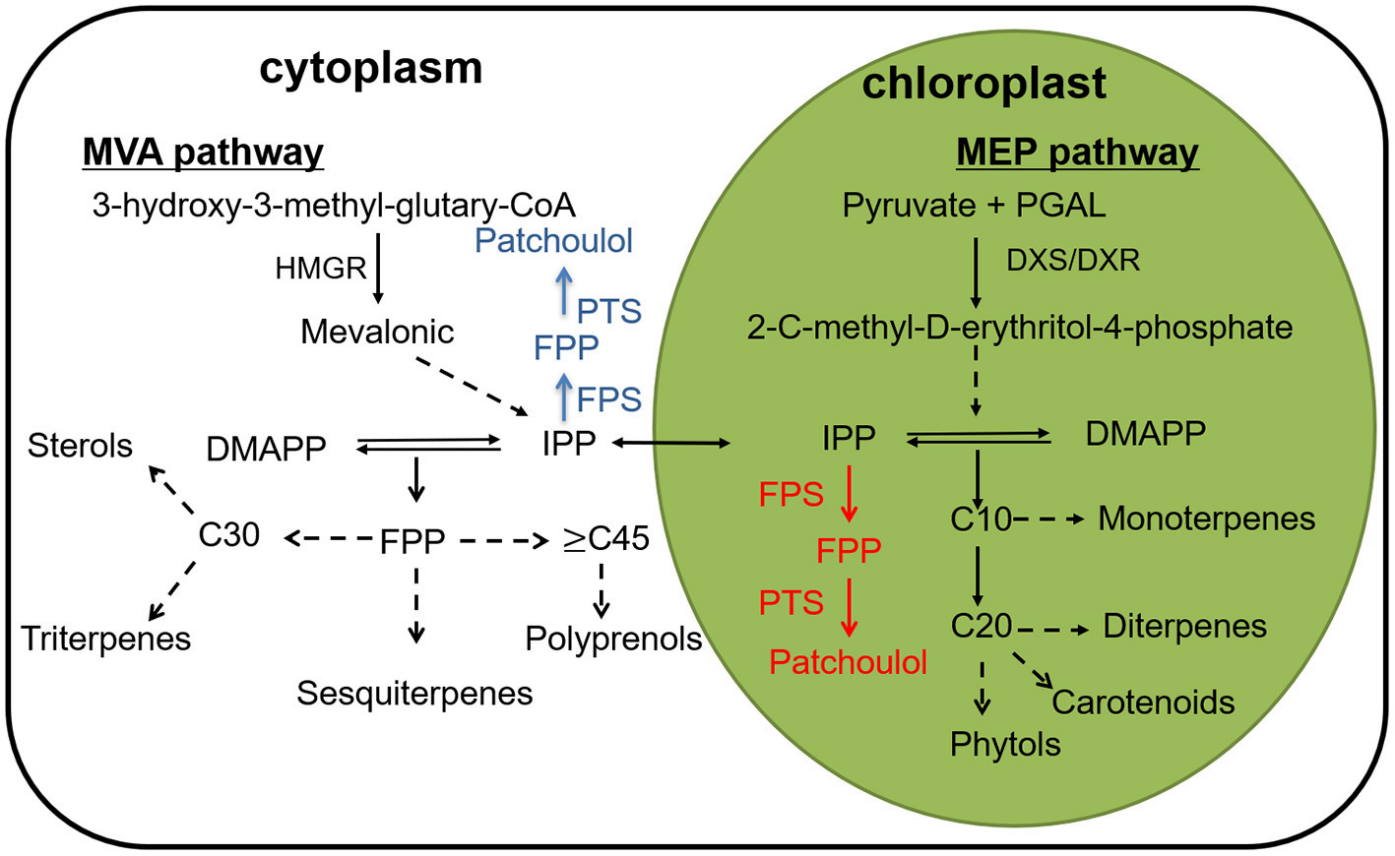
A depiction of the terpene biosynthesis, along with a conceptualization for patchoulol biosynthesis to the cytoplasm (blue) and to the chloroplast (red) compartments. HMGR, 3-hydroxy-3- methylglutaryl coenzyme A reductase; DXS, 1-deoxy-D-xylulose-5-phosphate synthase; DXR, 1-deoxy-D-xylulose5-phosphate reductase; FPS, farnesyl diphosphate synthase; FPP, farnesyl pyrophosphate; PTS, patchoulol synthase.

On the basis of the number of five-carbon (isoprene) units, terpenoids are classified into hemiterpenes (half-terpenes), monoterpenes (C_10_), sesquiterpenes (C_15_), diterpenes (C_20_), triterpenes (C_30_), tetraterpenes (C_40_), polyterpenes, and meroterpenes (Croteau, 2000). In plants, terpenoids are synthesized from two precursors, dimethylallyl diphosphate (DMADP) and isopentenyl diphosphate provided by MVA (mevalonate) and MEP (2-C-methyl-D-erythritol-4-phosphate) pathway (Rohmer et al., 1996; Rohmer, 1999; Lange et al., 2000). In plants, terpenoids are synthesized from two precursors, dimethylallyl diphosphate (DMADP) and isopentenyl diphosphate provided by MVA (mevalonate) and MEP (2-C-methyl-D-erythritol-4-phosphate) pathway (Daviet and Schalk, 2010). Afterwards terpene synthases catalyze the cyclization of GPP (geranyl diphosphate), FPP (farnesyl diphosphate) and GGPP (geranylgeranyl diphosphate) to generate the carbon skeletons of terpenoids. Finally, the enzymes further modify the terpene backbone to synthesize plenty of natural terpene derivatives, for example, cytochrome P450 monooxygenases (P450) (Cheng et al., 2007) (Figure 1).

Overexploitation and wasteful consumption of natural resources for high-value terpenes compounds, may drive the species to extinction and alter the environment. For instance, *Taxus chinensis* is famous for Taxol, an effective anti-cancer drug (Ru et al., 2006). However, *T. chinensis* is facing extinction because of deforestation (Zhang and Ru, 2010). To date, many efforts have been made to manipulate terpene metabolism in microorganism (Carter et al., 2003; Martin et al., 2003), fungus (Jackson et al., 2003; Ro et al., 2006; Westfall et al., 2012; Paddon et al., 2013), and plants (Wu et al., 2006; Farhi et al., 2011; Zhan et al., 2014; Wang et al., 2016) to synthetically produce more high-value chemicals.

Engineering terpene metabolism in plants is an innovative and attractive strategy to provide high-value terpenes. Plants have the elaborate biosynthetic ability and a cheaper way of using photosynthesis to produce high-value terpenes (Wu et al., 2006). For example, the metabolic engineered tobaccos stably transformed with the deregulated 3-hydroxy-3-methylglutaryl-coenzyme A reductase gene (*tHMG*), *ADS, CYP71AV1, CPR*, and artemisinic aldehyde reductase gene (*DBR2*), produced artemisinin, although the content in the transgenic tobaccos was much lower than that in *A. annua* plants (Farhi et al., 2011). In another study, the transgenic tobacco accumulated high-levels of terpenes, containing patchoulol, amorpha-4,11-diene, and limonene, via overexpressing farnesyl diphosphate synthase gene from avian and the terpene synthase gene to control the carbon flux (Wu et al., 2006). The results from these researches indicate the necessity of deeper exploring and demonstration of terpenes biosynthesis pathway genes with respect to over-expression strategies (Wu et al., 2006). In recent years, various strategies for engineering of triterpene squalene metabolism in tobacco have been developed. For instance, co-expression of farnesyl diphosphate synthase gene *FPS* from avian and yeast squalene synthase gene *SQS*, driven by trichome-specific gene promoter in the chloroplast, resulted in the accumulation of squalene to a high level in transgenic tobacco (Wu et al., 2012). These findings suggested that the accumulation of high-value terpenes could be observably elevated by directing the biosynthesis to other subcellular compartments.

Patchoulol, a volatile sesquiterpenoid isolated from leaves of *Pogostemon cablin* plants, is an important ingredient in fragrance products like perfumes, soaps and cosmetics. Plant patchouli is the only commercial source of this compound (Srikrishna and Satyanarayana, 2005). Limited natural resources lead to the fluctuation in the price of patchoulol between 30 and 200 US dollar/kg (Zhan et al., 2014). A sesquiterpene cyclase enzyme, patchoulol synthase was identified to catalyze FPP to form patchoulol in patchouli plants (Deguerry et al., 2006). With the development of molecular and synthetic biology, the engineered tobacco could produce 0.030 mg/g fresh weight (FW) patchoulol (Wu et al., 2006). Biotechnological production of patchoulol has been carried out in *Physcomitrella patens*, and the highest yield of patchoulol was 1.34 mg/g dry weight (Zhan et al., 2014). Besides, Albertsen et al. reported that expression of FPPS of yeast fused with PTS from *P. cablin* in *Saccharomyces cerevisiae* increased the production of patchoulol compared with the accumulation produced by PTS (Albertsen et al., 2011). Subsequently, several strategies were adopted to increase patchoulol content in *S. cerevisiae*. The shaken flask contained 59.2 ± 0.7 mg/L patchoulol, and a final production was 466.8 ± 12.3 mg/L (20.5 ± 0.5 mg/g dry cell weight) after fermentation optimization (Ma et al., 2019).

Here we demonstrated, an engineered heterogenous sesquiterpenoid patchoulol production in *A. annua*. The highest content of patchoulol was 52.58 μg/g dry weight in the transgenic plants by overexpressing farnesyl diphosphate synthase gene and patchoulol synthase gene. Furthermore, the accumulation of patchoulol was increased to 273 μg/g dry weight by altering the subcellular location of the introduced sesquiterpene synthetase expression.

## Result

### Patchoulol Was Produced in *FPS*+*PTS*-overexpressing Transgenic *A. annua* Plants

**Figure 2:**
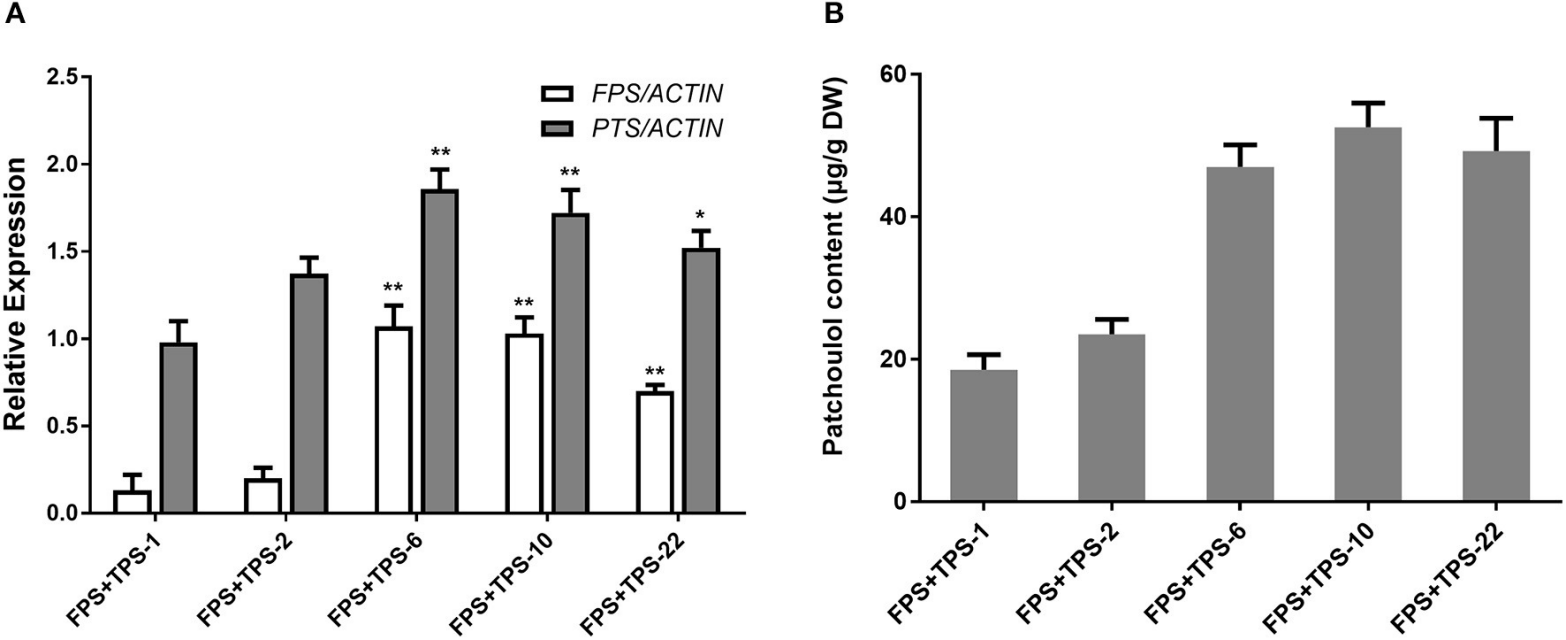
Engineering patchoulol biosynthesis in the cytoplasm of *A. annua* leaves. **(A)** Relative expression of *FPS* and *PTS* in *FPS*+*PTS* transgenic *A. annua* lines. **(B)** The patchoulol content in *FPS*+*PTS* transgenic *A. annua* lines. *ACTIN* was used as internal control. T0 transgenic lines were used for analysis. The error bars represent the means ± SD from three biological replicates. All data represent the means ± SD of three replicates. **P < 0.05, *P < 0.01, student's *t*-test.

To engineer the heterogenous sesquiterpenoid patchoulol production in *A. annua*, patchoulol synthase gene (*PTS*) from *P. cablin* and farnesyl diphosphate synthase gene (*FPS*) from an avian were chosen (Tarshis et al., 1994, Deguerry et al., 2006). Despite the cloned and identified *FPS* from *A. annua*, the avian *FPS* was observed not to be operated by the transcriptional or post-translational regulatory mechanisms in plants (Wu et al., 2006). To generate *FPS*+*PTS*-overexpressing transgenic *A. annua* plants, both *PTS* and *FPS* were transformed into *A. annua* plants via *A. tumefaciens* EHA105. Analysis of PCR showed that 25 independent lines were obtained. We then performed qRT-PCR to test the expression of both *FPS* and *PTS* genes in the transgenic lines. Among these, six transgenic lines showed a combination of high levels of *FPS* and *PTS* overexpression (Figure 2A). The patchoulol content in leaves was measured by GC-MS and the sesquiterpene alcohol patchoulol was successfully identified in *FPS*+*PTS*-overexpressing transgenic lines (Supplementary Figure 1). Quantification of patchoulol in transgenic lines showed that the six lines produced 23.51–52.58 μg patchoulol/g dry weight (Figure 2B), which was twice the transgenic tobaccos (Wu et al., 2006). The expressions of both *FPS* and *PTS* were higher than other transgenic lines in FPS+PTS-6 and 10, so they accumulated the highest patchoulol content.

### Repressing the Artemisinin Biosynthetic Pathway Is Useful for Getting a High Yield of Patchoulol

**Figure 3:**
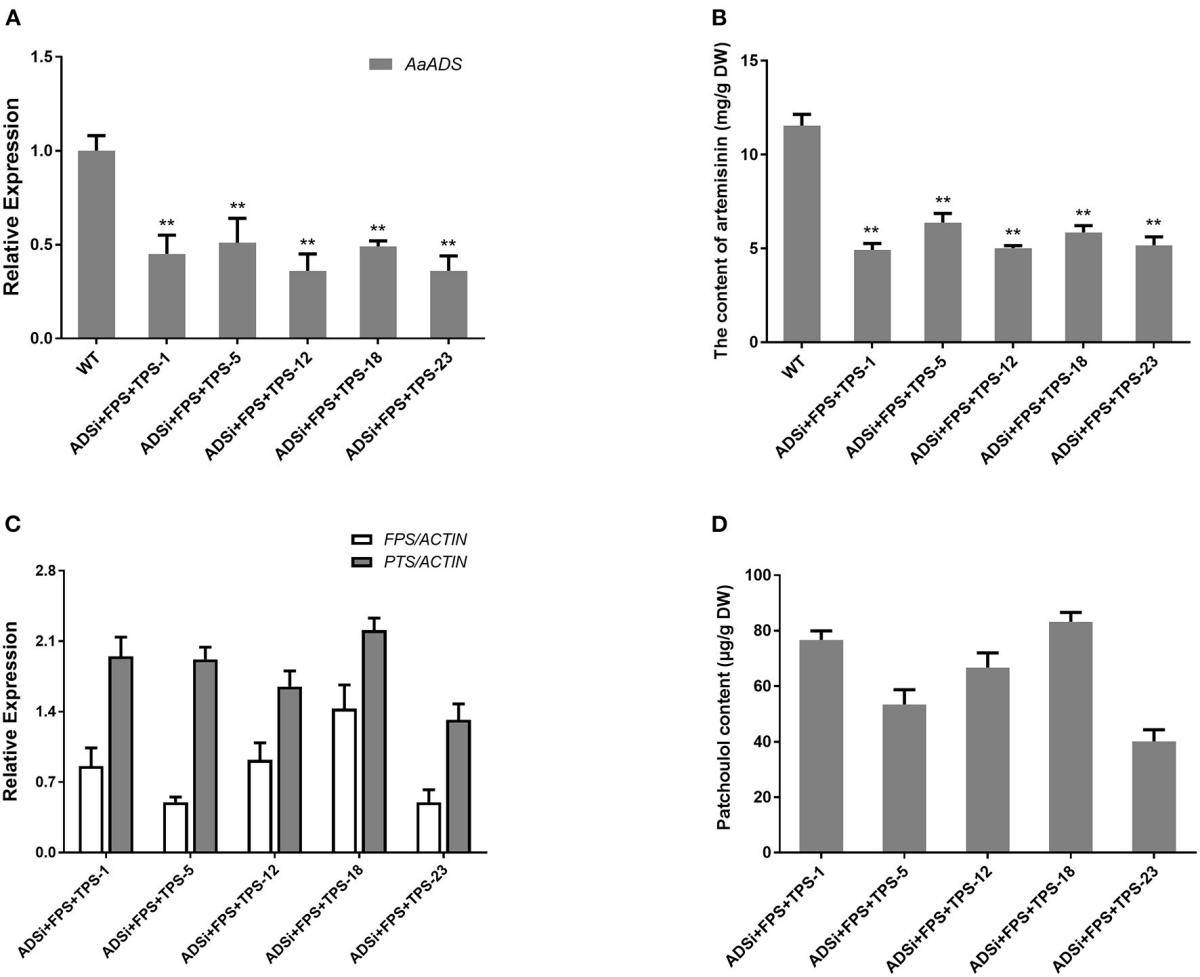
Blocking the artemisinin biosynthesis increased patchoulol content in *ADSi*+ *FPS*+*PTS* transgenic *A. annua* plants. **(A)** Relative expression of *ADS* in *ADSi*+ *FPS*+*PTS* transgenic *A. annua* lines. **(B)** The artemisinin content in *ADSi*+ *FPS*+*PTS* transgenic *A. annua* lines. **(C)** Relative expression of *FPS* and *PTS* in *ADSi*+ *FPS*+*PTS* transgenic *A. annua* lines. **(D)** The patchoulol content in *ADSi*+ *FPS*+*PTS* transgenic *A. annua* lines. T0 transgenic lines were used for analysis. *ACTIN* was used as internal control. The error bars represent the means ± SD from three biological replicates. All data represent the means ± SD of three replicates. **P < 0.05, *P < 0.01, student's *t*-test.

Blocking the competitive biosynthetic pathways is an effective approach for increasing the sesquiterpene content. Former studies have reported, diverse array of sesquiterpene synthases in *A. annua*. For sesquiterpene biosynthesis in *A. annua*, FPSs convert the common precursor FPP into an array of cyclized products, such as amorpha-4,11-diene via the action of ADS (Bouwmeester et al., 1999), β-caryophyllene by β-caryophyllene synthase (CPS) (Cai et al., 2002), β-farnesene by β-farnesene synthase (BFS) (Picaud et al., 2005), germacrene A by germacrene A synthase (GAS) (Bertea et al., 2006) and epi-cedrol by epi-cedrol synthase (ECS) (Mercke et al., 1999) respectively. Artemisinin, an important sesquiterpene isolated from *A. annua*, is ~0.1–1% of the dry weight in this plant, and artemisinin biosynthesis occurs in the cytosol in *A. annua* (Wallaart et al., 2001; Abdin et al., 2003). We speculated that silencing *ADS* gene competing for FPS with TPS by RNAi technology would enhance the patchoulol content in transgenic lines. To assess whether blocking the artemisinin biosynthetic pathway could increase the yield of patchoulol, the *FPS*+ *PTS* and *ADS*-RNAi constructs were co-transformed into *A. annua* plants resulting in 18 independent transgenic lines for further analysis. In transgenic lines, ADSi +FPS+ PTS −1, −5, −12, −18, and −23 had a combination of high levels of *FPS* and *PTS* transcripts (Figure 3C), as well as low *ADS* transcript level (Figure 3A). Compared with the wild-type, the artemisinin content was reduced to 42–55% in transgenic lines (Figure 3B). The results from patchoulol content GC-MS analysis showed that FPS+PTS+ADSi lines produced 41.13–83.23 μg patchoulol/g DW (Figure 3D). Consistent with the hypothesis, blocking the competing pathway could be an applicable approach for getting higher yield of patchoulol.

### The Localization of Heterologous Proteins

**Figure 4:**
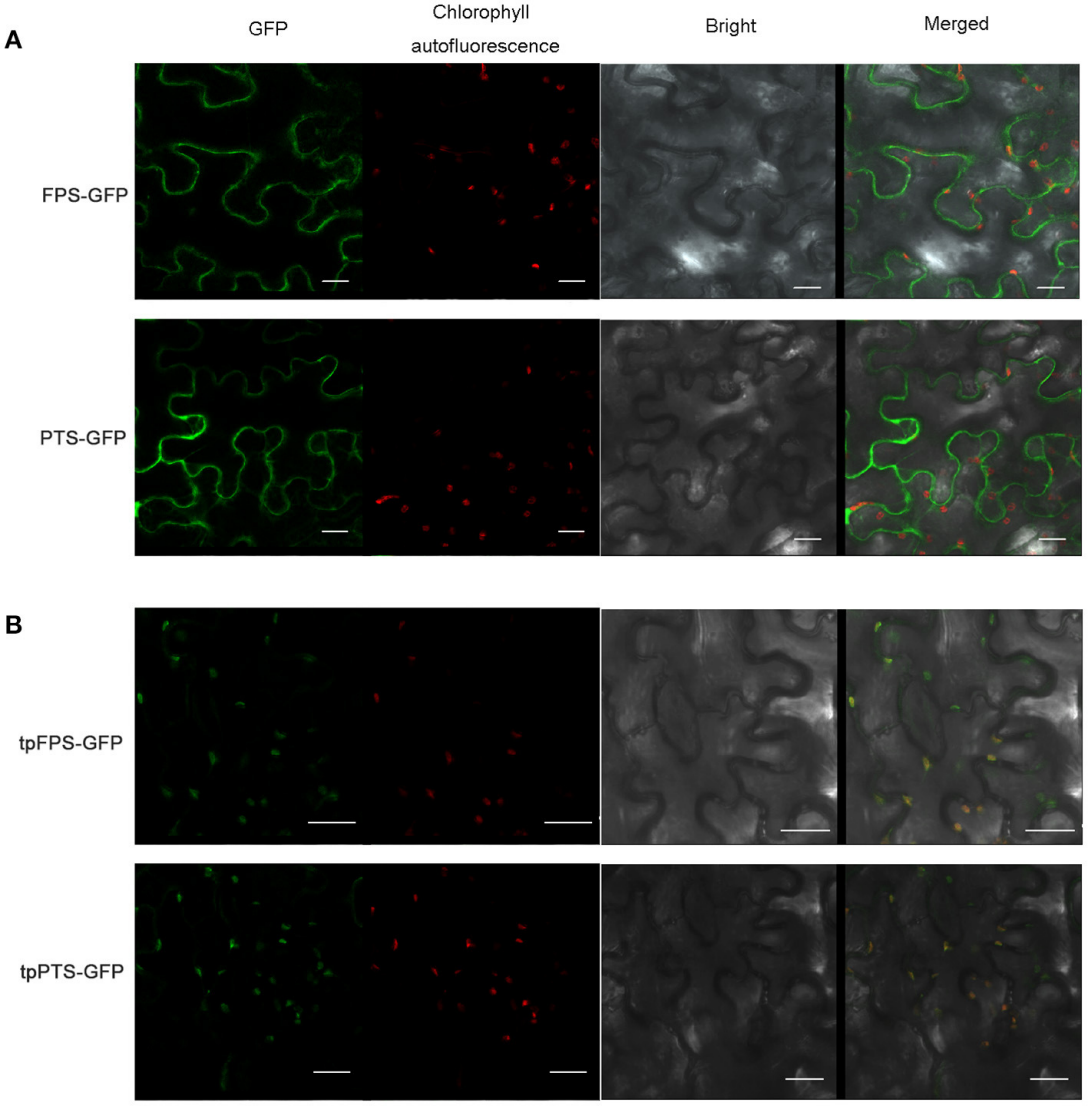
The subcellular localization of heterologous proteins. **(A)** Subcellular localization of FPS-GFP and PTS-GFP in tobacco leaf epidermal cells. **(B)** Subcellular localization of tpFPS-GFP and tpPTS-GFP in tobacco leaf epidermal cells. GFP: green fluorescent protein, Bars = 20 μm.

Many efforts had been made to introduce the terpene synthases into the cellular compartments, in which the terpene is naturally synthesized, to compete for substrates or overcome prospective rate-limiting steps. For instance, tobacco was used to produce heterologous patchoulol, in which, both *FPS* and *PTS* were expressed in tobacco, and the final yield was about 0.3 μg/g FW (Wu et al., 2006). Furthermore, higher level of patchoulol (30 μg/g FW) was observed in transgenic tobacco when chloroplast-targeting signal sequence from the signal peptide of *Arabidopsis* RUBISCO small unit (tpFPS and tpPTS) was fused with the amino terminus of both FPS and PTS (Lee et al., 2006; Wu et al., 2006). To confirm the localization of FPS and PTS, the full-length of *FPS* and *PTS* were fused with GFP (Green Fluorescent Protein), respectively. The recombinant plasmids were transiently expressed in tobacco leaves. The results showed that both FPS-GFP and PTS-GFP fusion proteins were separated from the fluorescence of chloroplasts (Figures 4A,B). When the chloroplast-targeting signal sequence (TP) was targeted to the amino terminus of both FPS and PTS, tpFPS-GFP and tpPTS-GFP fusion proteins were completely matched the fluorescence of chloroplasts (Figures 4A,B).

### Engineering the Patchoulol Biosynthesis in the Chloroplast Compartment Enhanced the Accumulation of Patchoulol

**Figure 5:**
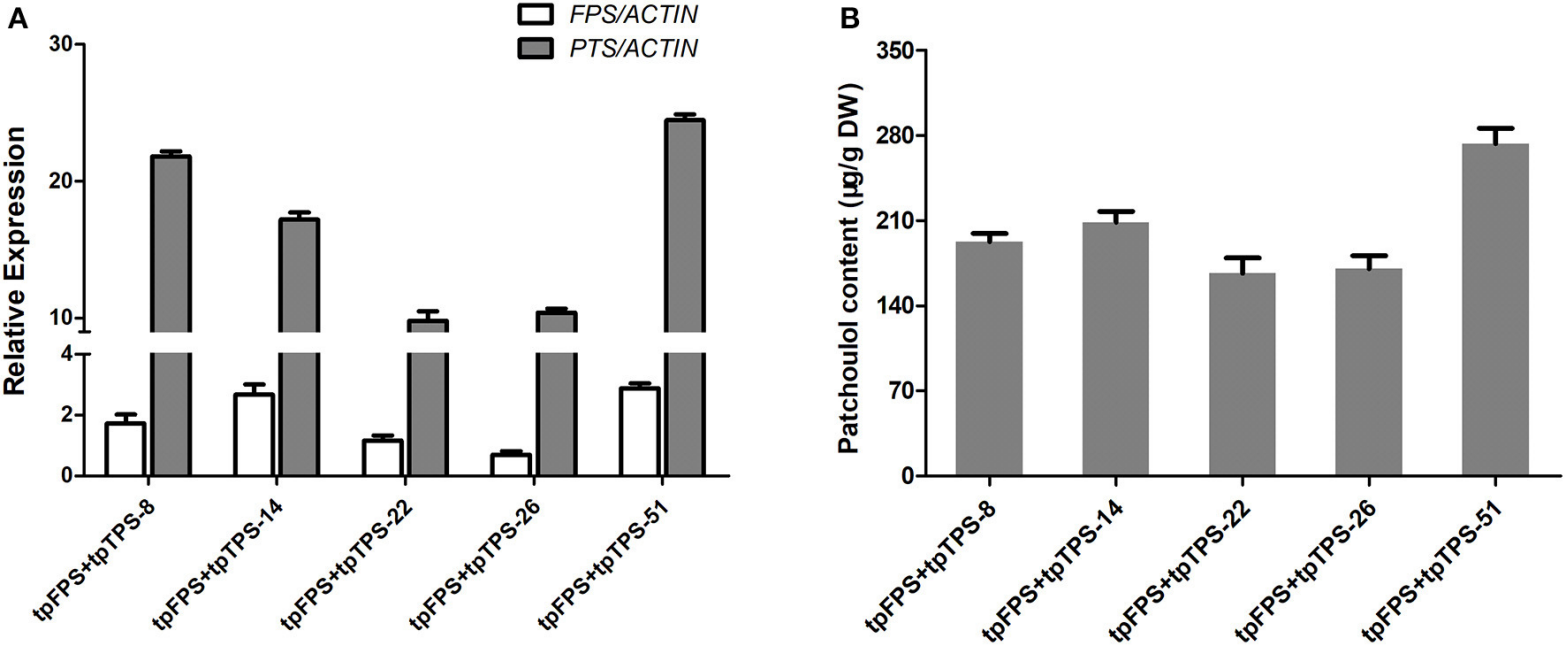
Engineering the patchoulol biosynthesis in the chloroplast compartment increased the patchoulol content in *tpFPS*+*tpPTS* transgenic *A. annua* plants. **(A)** Relative expression of *FPS* and *PTS* in *tpFPS*+*tpPTS* transgenic *A. annua* lines. **(B)** The patchoulol content in *tpFPS*+*tpPTS* transgenic *A. annua* lines. *ACTIN* was used as internal control. The error bars represent the means ± SD from three biological replicates.

When a particular terpene was produced in a certain compartment that this biosynthesis could not normally occur by diverting carbon flux at earlier intermediates, the high level of target product was obtained. For example, engineering of six genes encoding cytoplasmic MVA pathway, to chloroplast increased the levels of mevalonate, carotenoids, sterols, and squalene, suggesting the possible enhancement of overall terpene biosynthesis, despite of its organelles where it takes place (Kumar et al., 2011). Therefore, both FPS and PTS were further targeted into chloroplast via TP (tpFPS and tpPTS) (Lee et al., 2006) resulting in the more than 25 independent transgenic lines, which were further found to have higher expression levels of *tpFPS* and *tpPTS* (Figure 5A). The results from patchoulol content measurement by GC-MS revealed that, co-expression of *tpFPS* and *tpTPS* targeting the chloroplast compartment, could significantly enhance the patchoulol accumulation up to 273 μg/g DW (91 μg/g FW) (Figure 5B), which was 5–11-folds higher than those levels synthesized in the cytosol. The transgenic lines exhibited normal growth characteristics (Supplementary Figure 2).

## Discussion

Recently great efforts have been made to engineer terpene metabolism in plant (Lewinsohn et al., 2001; Wu et al., 2006, 2012; Zhan et al., 2014). *A. annua* is a traditional Chinese medicinal plant and is famous for artemisinin. A stable and efficient *Agrobacterium* mediated transformation system of *A. annua* has been established. Besides, there are a large amount of trichomes (glandular trichomes and T-shaped trichomes) on the leaves in *A. annua*, where large quantities of terpenes are synthetized and stored to protect plants against insects, pathogens, and herbivores (Wagner, 1991; Duke and Paul, 1993; Pichersky and Gershenzon, 2002). Recently the biochemistry of trichomes has been studied in *A. annua*. Numerous information from former studies on trichome development, provides a great opportunity for engineering of terpene biosynthesis in the specific target cellular compartment.

### The Advantage of Using *A. annua* as Platform for Synthetic Botany

In this work, we could apply and develop a novel approach and technique to engineer a cultivar of *A. annua*, with 273 μg/g DW patchoulol production with no any alteration of artemisinin biosynthesis. This strategy enables us to improve the economic value of medicinal plants. With the development of synthetic botany, many approaches have been made to produce valuable secondary metabolites. For instance, biosynthesis of β-carotene and anthocyanin in rice and the production of artemisinin in tobacco. However, due to the lack of specific storage cells in target plants, the heterogeneous biosynthesis of volatility chemicals, including mono- or sesquiterpene, still remain a big challenge in synthetic biology (Houshyani et al., 2013).

For instance, Wu et al. have constructed the patchoulol biosynthesis pathway in the tobacco plastid with the same strategy used in this study. However, the patchoulol content in the best-performing transgenic tobacco line was reported to be 30 μg/g FW, while the highest patchoulol content in transgenic *A. annua* in our study, reached to the level of 273 μg/g DW (91 μg/g FW) which could be possibly resulted from the possession of numerous glandular trichomes on the epidermal cells of *A. annua* leaves, where the accumulation and storage of artemisinin and lots of other mono- or sesquiterpene takes place. Furthermore, there is no significant difference in the artemisinin contents between patchoulol produced in transgenic and wild type *A. annua* (Supplementary Figure 3), suggesting the transgenic *A. annua* to be a reliable sources of anti-malaria agent, artemisinin and flavor component patchoulol production. Beside the above evidences, the establishment of an efficient *Agrobacterium* mediated transformation system in *A. annu*, gives a noticeable credit to it for being a worthy candidate in biosynthetic biology.

### The Enzyme Targeted Cellular Compartment Is Crucial for Patchoulol Biosynthesis in *A. annua*

It has been well-studied that the terpenoids are biosynthesized from two independent compartmentally pathways: the MVA and MEP pathways. The MEP pathway, located in plastid, is dominantly responsible for the biosynthesis of mono- and diterpenes. The cytoplasm located MVA pathway is mainly responsible for the biosynthesis of sesquiterpenes. In *A. annua*, the artemisinin biosynthesis depends on cytoplasm directed MVA pathway (Newman and Chappell, 1999; Weathers et al., 2006). However, only about 0.001% dry weight of dihydroartemisinic alcohol (the artemisinin precursor) is reported to be produced in tobacco when expressing the artemisinin biosynthetic genes in the cytoplasm. On the contrast, the artemisinic acid accumulation reached to a maximum of about 0.004% when transferring the entire artemisinin biosynthetic genes into the chloroplast (Saxena et al., 2014). Wu et al. also reported that targeting the *PTS* to the plastid increased the patchoulol accumulation as well. In this study, targeting *PTS* into *A. annua* plastid produced higher patchoulol compared to its expression in cytoplasm. These results suggested that targeting or expressing the enzymes in plastid might be a powerful tool for synthetic botany.

### Pathway Block Is a Useful Method for Improving Patchoulol Accumulation

Besides higher production and accumulation of patchoulol through targeting the *PTS* into *A. annua* plastid, we also investigated the influence of pathway blockage toward the patchoulol biosynthesis in cytoplasm. Obviously, the risk will be risen when the metabolic pathway is composed of more than two genes. For improving the patchoulol yield in cytoplasm, we blocked the artemisinin biosynthesis through RNA interference for *ADS* gene. The results showed that the patchoulol content had significantly increased in the ADSi lines affirming the positive role of pathway blockage in elevation of patchoulol biosynthesis. However, the patchoulol content in ADSi+PTS+FPS lines was still lower than that in transgenic plastid targeted *PTS* in *A. annua*, which could be the result of incomplete blockage of artemisinin biosynthesis by RNA interference. In addition, the presence of other competitive pathway along with artemisinin biosynthesis could lead to the deficiency of RNAi for higher production of patchoulol.

## Materials and Methods

### Plant Material and Growth Conditions

*A. annua* seeds originated from Chongqing province, were developed in Shanghai. *A. annua* plants were cultured in the greenhouse (16/8 h day/night, 25°C). Tobacco (*Nicotiana benthamiana*) was grown under the same conditions as *A. annua*.

### Vectors Construction and the Transformation of *A. annua*

The patchoulol synthase gene (*PTS*) from *P. cablin* and farnesyl diphosphate synthase (*FPS*) from an avian were, respectively, inserted into the AscI/XhoI and SpeI/KpnI sites of the helper pTDUA vector. For the plastid-targeted expression, the transit peptide signal sequence of the RUBISCO protein in *Arabidopsis* (GenBank accession NM23202) was added to the 5' end of *FPS* and *PTS*, respectively. *PTS* driven by the cassava mosaic promoter and *FPS* driven by the 35S promoter were subsequently transferred to the pDONR vector through Gateway recombination reaction. The expression vectors were provided by Firmenich.

The 300 bp fragment of *AaADS* (GenBank accession AF138959) was cloned into the intermediate cloning vector pDONR, and transferred to the pHELLSGATE12 vector by LR recombination reaction (Invitrogen, Carlsbad, CA, USA). The information about the primers used are listed in Supplementary Table 1. The recombinant plasmids were transferred into *Agrobacterium tumefaciens* strain EHA105, then used to introduced into *A. annua* plants (Zhang et al., 2009). After 3–4 months, the regenerated plants were obtained.

### Transcript Analysis of Terpene Synthase Genes

The first leaves were collected from 3 month-old *A. annua* plants for RNA extraction. Total RNA was extracted using the RNAprep Pure Plant Kit (Tiangen, Beijing, China). The first-strand cDNA for qRT-PCR was synthesized using the PrimeScript II first Strand cDNA Synthesis Kit (Takara, Shiga, Japan). RT-qPCR was performed using SuperReal PreMix Plus (Tiangen, Beijing, China). β*-ACTIN* was used as the reference gene. RT-qPCR was performed in a Roche LightCycler96 (Roche). According to the manufacturer's instructions, amplification was carried out using SYBR Green qPCR MasterMix (Takara, Shiga, Japan). The profile for SYBR Green qPCR was 95°C for 10 min, followed by 40 cycles of 95°C for 20 s, 55°C for 20 s, and 72°C for 20 s. The primers are represented in Supplementary Table 1. Three biological repeats were measured for each sample. The transcripts level was calculated using the 2^−Δ*Ct*^ method. *ADS* expression was analyzed using 2^−Δ*ΔCt*^ (Kilambi et al., 2013).

### Subcellular Localization

To determine the subcellular localization of FPS and PTS proteins, the green fluorescent protein (GFP) was fused to the C-terminal domain of FPS, PTS, tpFPS, and tpPTS under the control of the CaMV35S promoter. The recombinant plasmids (pHB-FPS-GFP, pHB-PTS-GFP, pHB-tpFPS-GFP, and pHB-tpPTS-GFP) were, respectively, transferred into GV3101. The strains GV3101 harboring the recombinant plasmids were, respectively, co-transformed into *N. benthamiana* leaves with the strain containing p19 plasmid (Voinnet et al., 2003). GFP signals were observed by confocal laser microscopy (Leica TCS SP5-II) after 48 h incubation.

### Quantification of Artemisinin by HPLC-ELSD

Quantification of artemisinin was performed as described previously (Zhang et al., 2009). Leaves from 3 month-old *A. annua* were collected, dried for 48 h at 50°C and pulverized into powder. 0.1 g dried-leaf powder was used for the ultrasonic extraction with 1 mL methanol for 30 min. Then the mixture was centrifuged at 12, 000 rpm for 5 min. The supernatants were filtered using filters (0.22 μm). The samples were analyzed by the HITACHI *2695* HPLC system coupled with a SANCO ELSD180 detector. The conditions were as follows: mobile phase, water/methanol (20:80, v/v); column, YMC-Pack ODS-A C18; flowrate, 1 ml/min. Artemisinin was set at 5.577 for artemisinin. The artemisinin standard was purchased from Sigma. Three biological repeats were measured for each sample.

### Quantification of Patchoulol by GC-MS

Quantification of patchoulol was identified and quantified using gas chromatography and mass spectrometry (GC-MS). Five hundred milligram leaves were collected from 3 month-old *A. annua* and rapidly ground into powder in liquid nitrogen. The powder was used for the ultrasonic extraction with 3 ml ethyl acetate for 20 min. Then the samples were centrifuged at 5,000 g for 10 min. And the supernatants were filtered using filters (0.22 μm). Meanwhile, 500 mg leaves of *A. annua* were collected and dried at 60°C overnight. The weights were accurately measured for the calculation of the dry weight. Quantification was achieved based on the standard patchoulol (Aladdin, China). GC-MS analysis was performed on a GC-MS 7890/5975C (Agilent) according to the methods described previously (Zhan et al., 2014). Dodecane was used as the internal standards.

## Data Availability Statement

The raw data supporting the conclusions of this article will be made available by the authors, without undue reservation.

## Author Contributions

XF and KT designed the research. XF and FZ performed the experiments and wrote the first draft of the manuscript. YM, BP, QP, ZD, WL, JZhang, JZhao, and XS analyzed the data. XF, KT, and DH drafted the manuscript. YZ, DC, and LL revised the manuscript. All authors approved the manuscript.

## Conflict of Interest

YZ, ZD, WL, JZhang, LH, and DC were employed by company Firmenich Aromatics (China) Co. Ltd. The remaining author declares that the research was conducted in the absence of any commercial or financial relationships that could be construed as a potential conflict of interest.
