# Supplementary material for: High-Level Patchoulol Biosynthesis in Artemisia annua L
Source: Front Bioeng Biotechnol. 2021 Feb 4;8:621127. doi: 10.3389/fbioe.2020.621127 (PMC7890116; doi:10.3389/fbioe.2020.621127)
Supplement: Supplementary file 1 [file Data_Sheet_1.PDF]

S1 Table. Primers used in this study.

No.	Primer name	Use	Primer sequence 5´-3´
1	FPS-FP	PCR	ATGCAGCCCCATCATCATCATA
2	FPS-RP	PCR	GGGTCCCCAAAGCAGTCCAGGTAA
3	TP-FP	PCR	ATGGCTTCCTCTATGCTCTC
4	PTS-FP	PCR	ACATCACTTCCATCGCAAGCAACG
5	PTS-RP	PCR	ACATCACTTCCATCGCAAGCAACG
6	FPS-QPCR-FP	qPCR	GAAGGATGCTGAGAGCCTGCGGTG
7	FPS-QPCR-RP	qPCR	ATGAGGTCCAGCATCTGCCCGAGC
8	TPS-QPCR-FP	qPCR	GATTGGGTGTTCTCCCGACCTCCT
9	TPS-QPCR-RP	qPCR	TCTTTGTAAATAACCTCAAGTGTTCGGA
10	β-actin-FP	qPCR	CCAGGCTGTTCAGTCTCTGTAT
11	β-actin-RP	qPCR	CGCTCGGTAAGGATCTTCATCA
12	ADS-RNAi-FP	cloning	CCTATTCGCCCCATTGCCAACTT
13	ADS-RNAi-RP	cloning	CGGAACCATAAGGAAGAGCGGT
14	ADS-QPCR-FP	qPCR	AAGCAAGTAGAGCAAGGGGTGGAAC
15	ADS-QPCR-RP	qPCR	TGCTTGAACGCTCCATTTTTGTCTT
16	pHellsgate-35S-FP	PCR	CGAAAGGACAGTAGAAAAGGAAGGTGGC
17	FPS-BamHI-GFP-FP	cloning	CTCAAGCTTGGATCCATGCAGCCCCATC ATCA TCATAAAG
18	FPS-SpeI-GFP-RP	cloning	GCTCACCATACTAGTTTTTCTGGCGTTTG TAGA TCTTC
19	BamHI-TPS-GFP-FP	cloning	CTCAAGCTTGGATCCATGGCTTCCTCTA TGCT CTCCT
20	SpeI-TPS-GFP-FP	cloning	GCTCACCATACTAGATATGGAACAGGG TGAA GGTAC
21	BamHI-TP-GFP-FP	cloning	CTCAAGCTTGGATCCATGCAGCCCCATC ATCA TCATA

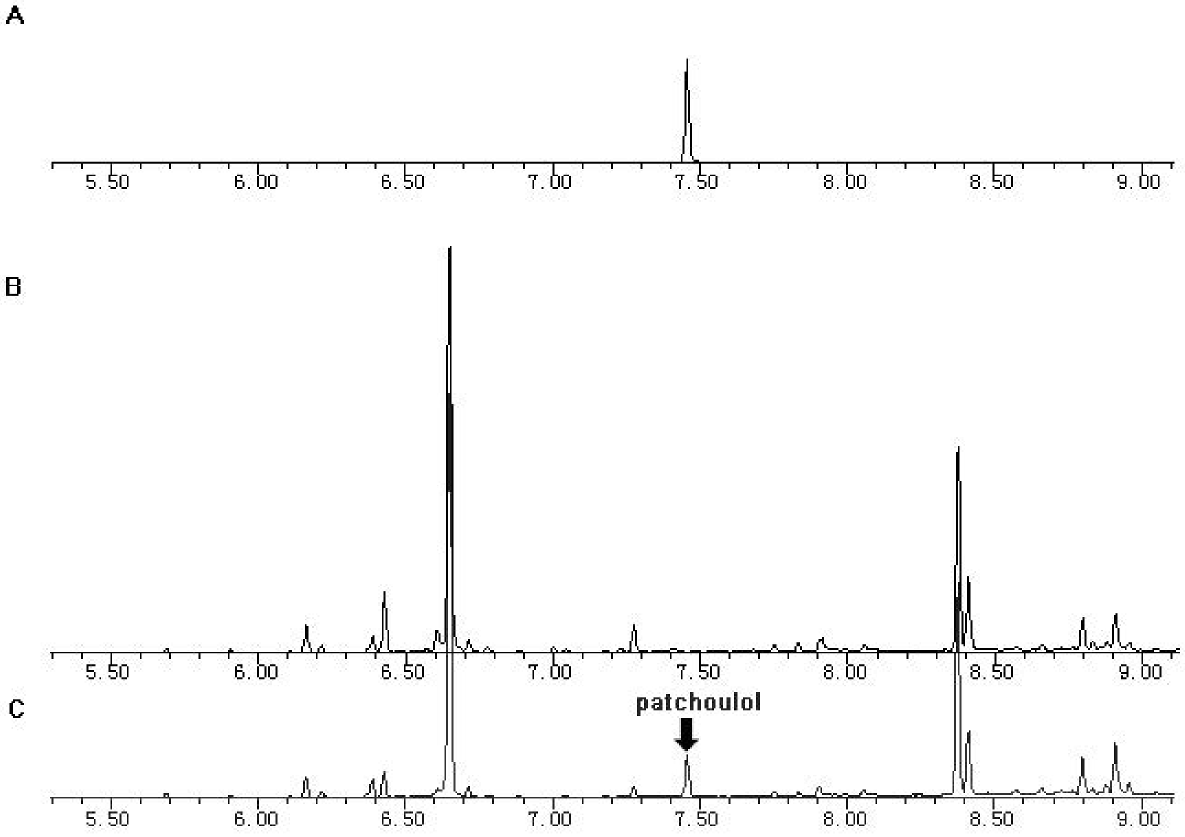

Figure S1 The patchoulol analysis was carried out by GC-MS.

A.The patchoulol standard. B. Analysis of leaves extracts from the wild type *A. annua* plant. C. Analysis of leaves extracts from the transgenic *A. annua* plant.

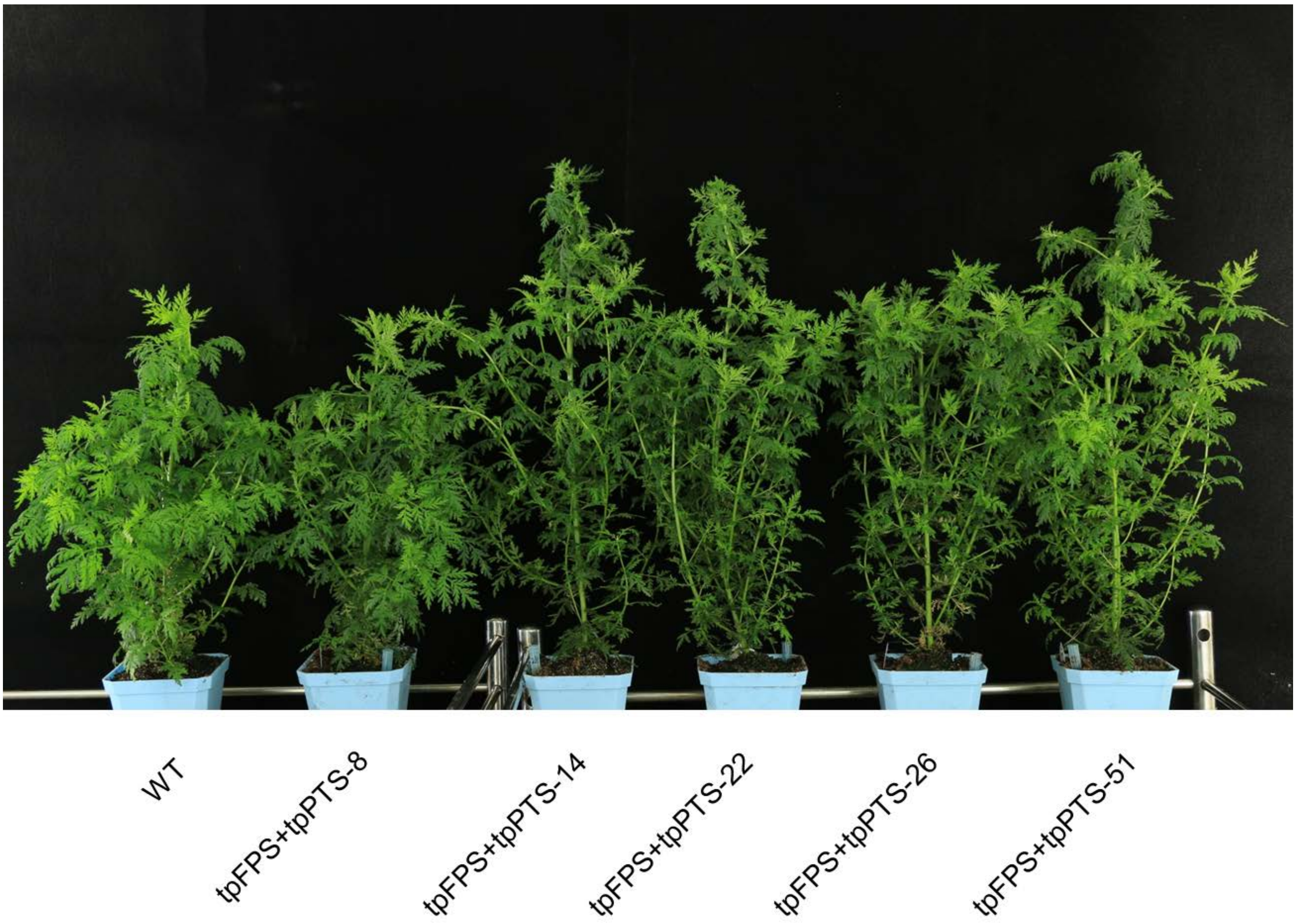

Figure S2 Wild type and *tpFPS+tpPTS* transgenic *A. annua* plants.

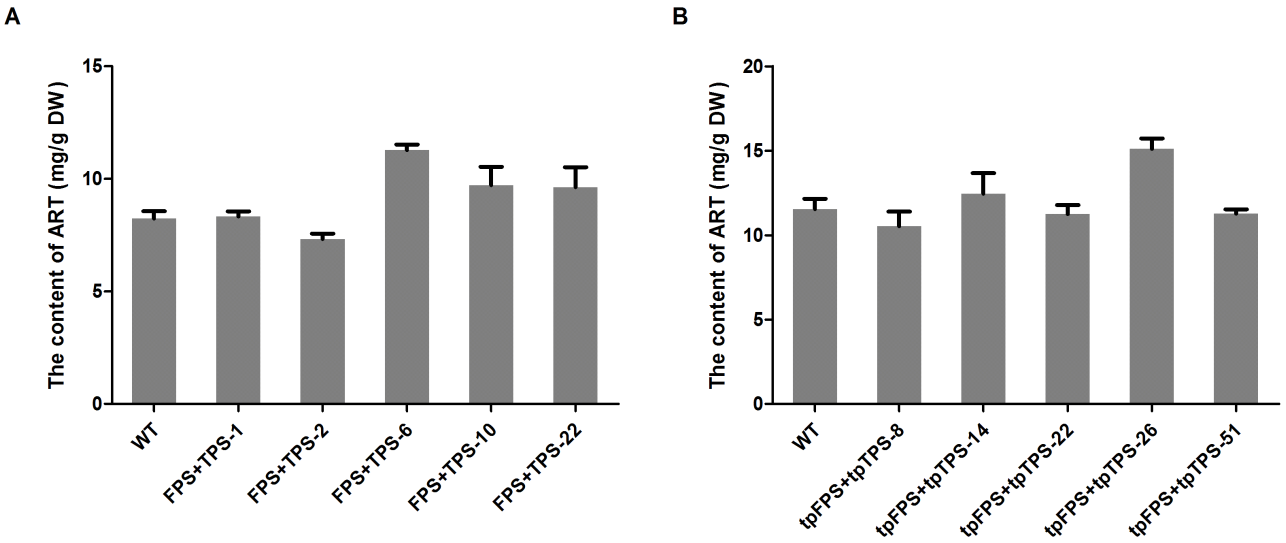

Figure S3 The artemisinin contents were analyzed by HPLC.

A.The artemisinin content in *FPS+PTS* transgenic *A. annua* lines. B. The artemisinin content in *tpFPS+tpPTS* transgenic *A. annua* lines. The error bars represent the means ± SD from three biological replicates.

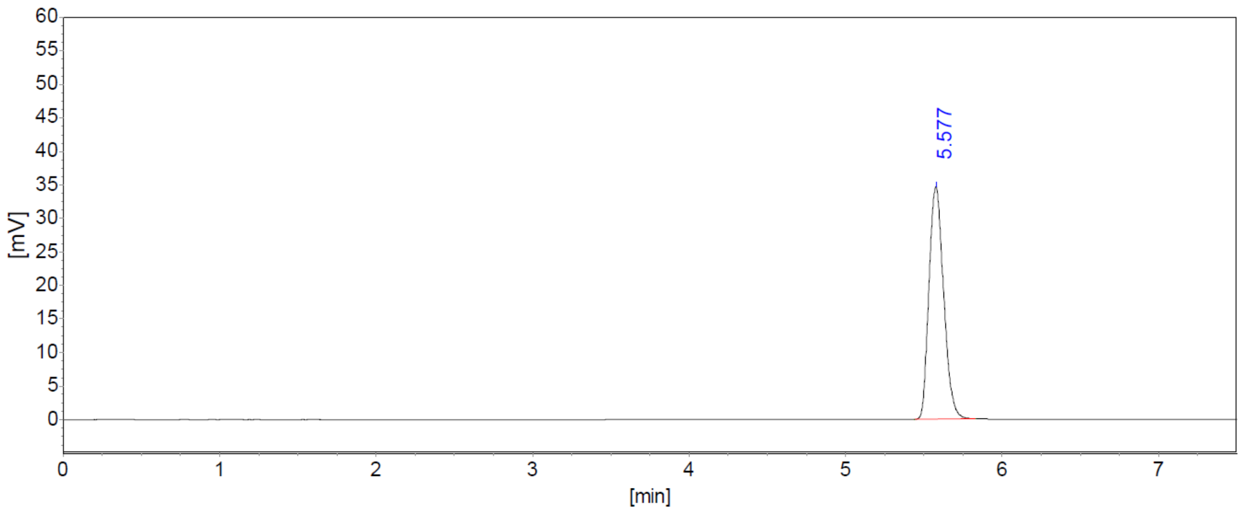

Figure S4 The chromatogram of artemisinin standard.
The artemisinin standard is analyzed by HPLC.
